# ANXIETY IN PATIENTS UNDERGOING OPEN-HEART SURGERY: THE ROLE OF CHARACTER, STRENGTH, AND HOPE IN A STRUCTURE EQUATION MODEL

**DOI:** 10.1093/geroni/igad104.2529

**Published:** 2023-12-21

**Authors:** Veronika Nash, Amy Ai

**Affiliations:** Florida State University, Tallahassee, Florida, United States; Florida State University, Tallahassee, Florida, United States

## Abstract

In an era of patient-centered care, more research is needed to examine the protective function of patients’ internal strengths against the damage of poor mental health. Mental health plays a significant role in cardiac health. Anxiety is a mortality risk for cardiac patients, the evidence has indicated high prevalence (13-59.5%) post cardiac events. However, anxiety hasn’t been extensively investigated as depression in cardiac patients, especially after open-heart surgery (OHS). Even less is known about protective function of Character Strength Hope in cardiac anxiety during stressful OHS, also a life-altering intervention for advanced heart conditions. This interdisciplinary study evaluated a structural equation model (SEM) on the relationship of Hope with pre- and post-OHS anxiety. Two weeks prior to the scheduled operation, research assistants conducted a face-to-face psychosocial interview with 481 patients (male, 58%; mean age=62 years, range=35-89) at the hospital, followed by a second telephone interview to assess an indicator of Hope two-days prior to surgery. Postoperative measures were assessed 36 days after OHS. Female and older patients reported higher levels of anxiety preoperatively. The SEM demonstrated adequate fit of the final solution in which pre- and post-OHS anxiety levels were related. Higher levels of pre-OHS anxiety were related to lower levels of optimism and hope right before OHS. Higher levels of optimism, indicating Hope predicted lower levels of post-OHS anxiety. Because patients with high anxiety would be disadvantaged in postoperative recovery, our finding suggests enhancing preoperative strength, Hope, might have some psychological benefits for optimal outcomes in patients undergoing OHS.

